# Regional variation and determinants of vitamin D status in sunshine-abundant Thailand

**DOI:** 10.1186/1471-2458-11-853

**Published:** 2011-11-10

**Authors:** La-or Chailurkit, Wichai Aekplakorn, Boonsong Ongphiphadhanakul

**Affiliations:** 1 Department of Medicine, Ramathibodi Hospital, Mahidol University, Bangkok 10400, Thailand; 2 Department of Community Medicine, Ramathibodi Hospital, Mahidol University, Bangkok 10400, Thailand; 3 Division of Endocrinology and Metabolism, Department of Medicine, Faculty of Medicine Ramathibodi Hospital, Mahidol University, Rama 6th Road, Bangkok 10400, Thailand

## Abstract

**Background:**

Vitamin D insufficiency is highly prevalent. Most of the studies concerning vitamin D status were generated from countries situated at temperate latitudes. It is less clear what the extent of vitamin D insufficiency is in countries situated in the tropics and how geographical regions within country would affect vitamin D status. In the present study, we investigated vitamin D status in Thais according to geographical regions and other risk factors.

**Methods:**

Subjects consisted of 2,641 adults, aged 15 - 98 years, randomly selected from the Thai 4th National Health Examination Survey (2008-9) cohort. Serum 25 hydroxyvitamin D were measured by liquid chromatography/tandem mass spectrometry. Data were expressed as mean ± SE.

**Results:**

Subjects residing in Bangkok, the capital city of Thailand, had lower 25(OH)D levels than other parts of the country (Bangkok, central, northern, northeastern and southern regions: 64.8 ± 0.7, 79.5 ± 1.1, 81.7 ± 1.2, 82.2 ± 0.8 and 78.3 ± 1.3 nmol/L, respectively; *p *< 0.001). Within each region, except for the northeastern part of the country, subjects living inside municipal areas had lower circulating 25(OH)D (central, 77.0 ± 20.9 nmol/L vs 85.0 ± 22.1 nmol/L, *p *< 0.001; north 79.3 ± 22.1 nmol/L vs 86.8 ± 21.8 nmol/L, *p *< 0.001; northeast 84.1 ± 23.3 nmol/L vs 87.3 ± 20.9 nmol/L, *p *= 0.001; south, 76.6 ± 20.5 nmol/L vs 85.2 ± 24.7 nmol/L, *p *< 0.001). Overall, the prevalence of vitamin D insufficiency was 64.6%, 46.7%, and 33.5% in Bangkok, municipal areas except Bangkok, and outside municipal area in other parts of the country, respectively. In addition, the prevalence of vitamin D insufficiency according to geographical regions was 43.1%, 39.1%, 34.2% and 43.8% in the central, north, northeast and south, respectively. After controlling for covariates in multiple linear regression analysis, the results showed that low serum 25(OH)D levels were associated with being female, younger age, living in urban and Bangkok.

**Conclusions:**

Vitamin D insufficiency is common and varies across geographical regions in Thailand.

## Background

Vitamin D is produced endogenously when the skin is exposed to sunlight, or obtained exogenously from nutrients or supplements. The major role of vitamin D is to maintain calcium homeostasis and bone health. In addition, recent research has revealed that vitamin D may play an important role in a variety of non-skeletal health activities, such as modulation of neuromuscular and immune function, reduction of inflammation, and regulation of cell proliferation, differentiation and apoptosis. Vitamin D insufficiency is highly prevalent and is now recognized as a worldwide health problem [1-3]. It has been observed to varying degrees in many different countries, regardless of geographical location [4]. However, most of the studies concerning vitamin D status have focused on countries situated at temperate latitudes. It is less clear what the extent of vitamin D insufficiency is in countries situated in the tropics, and how geographical regions within a country could affect vitamin D status.

Thailand, a Southeast Asian country, is located at latitudes between 5°30' N and 20°30' N. Vitamin D intake among Thais is generally low because few natural vitamin D-rich food sources are found in Thailand, and foods are not fortified with vitamin D. Up to now, there has been a lack of reliable epidemiological data concerning vitamin D status in Thais. Therefore, the purpose of this study is to investigate vitamin D status in Thais according to geographical region by assessing levels of serum 25-hydroxyvitamin D (which is the major metabolite and represents the stored form of vitamin D) by a reference method, liquid chromatography/tandem mass spectrometry (LC-MS/MS).

## Subjects and methods

### Population

**Figure 1:**
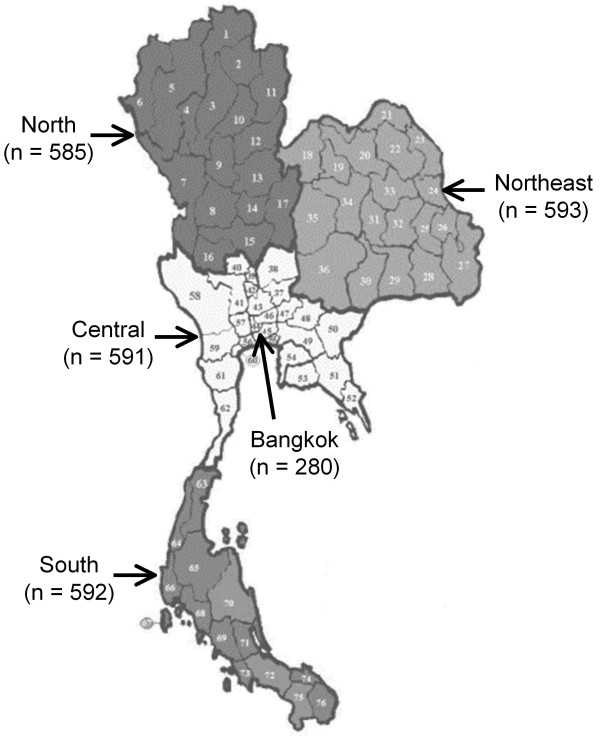
**Geographical regions of the study**.

This study used data from the Thai 4th National Health Examination Survey (NHESIV) conducted between August 2008 to March 2009 by the National Health Examination Survey Office, Health System Research Institute. Subjects aged 15-98 years were randomly selected from 21 provinces in four geographical regions of Thailand as well as the capital city, Bangkok (Figure 1) using stratified, multistage probability sampling of the population aged ≥15 years with a sample size of 21,960 individuals. The skin color of Thais are categorized into skin types 4 (Brown)-5(Dark Brown) by the Fitzpatrick Classification Scale, both of which possess the high capability to produce melanin. Demographic data such as age, sex, and religion were included. Body weight and height were measured using standard procedure. Body mass index (BMI) was calculated as weight in kilograms divided by the square of height in meters. Fasting blood samples were obtained and transferred to a freeze at a central laboratory in Ramathibodi Hospital, a university hospital Bangkok, where they were kept at -80°C. The present study used a subsample of the NHES-IV serum samples to measure serum levels of 25-hydroxyvitamin D (25(OH)D). The subsamples were randomly selected according to age group (15-29, 30-44, 45-59, 60-69, 70-79, and ≥80 years), sex, urban/rural and region. In each stratum, 25 individuals were randomly selected using statistical software. A total of 2,700 were sampled of which 2,641 serum samples were available. The study was approved by the ethics committee of Ramathibodi Hospital. Informed consent was obtained from all subjects.

### Serum 25-hydroxyvitamin D (25(OH)D) measurement

Serum 25(OH)D2 and 25(OH)D3 were analyzed by LC-MS/MS with an Agilent 1200 Infinity liquid chromatograph (Agilent Technologies, Waldbronn, Germany) coupled to a QTRAP^® ^5500 tandem mass spectrometer (AB SCIEX, Foster City, CA, USA) using a MassChrom^® ^25-OH-Vitamin D3/D2 diagnostics kit (Chromsystems, Munich, Germany). The summation of serum 25(OH)D2 and 25(OH)D3 was used to reflect vitamin D status. The inter-assay and intra-assay coefficients of variation of total serum 25(OH)D level were 6.3% and 5.0%, respectively.

### Statistical analysis

Data were expressed as mean ± SE. Differences between two groups were assessed by Student's *t*-test. Comparisons among three or more groups were analyzed by analysis of variance followed by Scheffé's test. Stepwise multiple linear regression analysis was used to examine the independent determinants of variables. A *p *value less than 0.05 was considered statistically significant. All analyses were performed using Stata version 10.1 (StataCorp LP, Texas, USA) and SPSS statistical software, version 16.0 (SPSS Inc., Chicago, IL, USA). All the data analyses were weighted to the probability of sampling to take into account for complex survey design.

## Results

**Figure 2:**
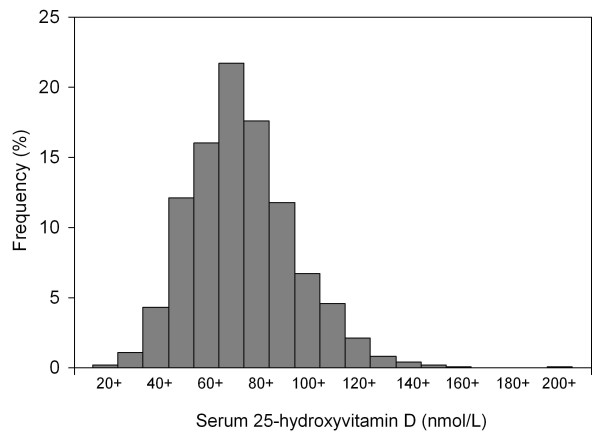
**Frequency distribution of 25-hydroxyvitamin D levels in Thais**.

**Figure 3:**
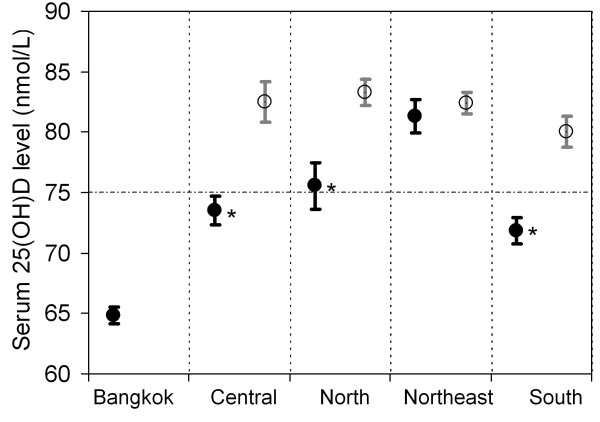
**Vitamin D status inside (closed circle) and outside (open circle) the municipal area in each region**. Values are mean ± SE. * = significant compared to outside the municipal area within region

The Baseline characteristics of the population study were shown in Table 1. The frequency distribution of serum 25(OH)D levels was shown in Figure 2. Data on average duration of sunlight (Table 2) and temperature in Thailand were obtained from the Thai Meteorological Department, for year 2008 and 2009. The minimum and maximum temperature ranged from 6.0°C to 42.4°C. Mean serum 25(OH)D levels in Thais according to geographical region are shown in Table 2. Subjects residing in Bangkok had lower 25(OH)D levels than those in other parts of the country, and the mean value was also below the sufficient level (75 nmol/L); whereas subjects residing in the northeastern region had the highest mean serum 25(OH)D level. Within each region, except for the northeastern part of the country, subjects living inside municipal areas had lower circulating 25(OH)D: central, 73.5 ± 1.2 nmol/L vs 82.5 ± 1.7 nmol/L, *p *< 0.001; north 75.6 ± 1.9 nmol/L vs 83.3 ± 1.1 nmol/L, *p *< 0.001; northeast 81.3 ± 1.4 nmol/L vs 82.4 ± 0.9 nmol/L, *p *= 0.001; south, 71.9 ± 1.1 nmol/L vs 80.1 ± 1.3 nmol/L, *p *< 0.001 (Figure 3). When only municipal areas were analyzed, subjects in Bangkok still had significant lower 25(OH)D levels than the rest of the country (*p *< 0.01) (Figure 3). In addition to geographical region, there were significant differences in mean serum 25(OH)D levels by gender, age, living in municipal areas, BMI status and religion (Table 2 and Table 3). Moreover, lower serum 25(OH)D levels was observed in younger age (Table 3). Overall, the prevalence of vitamin D insufficiency, as defined by 25(OH)D levels less than 75 nmol/L, in Bangkok was 64.6%; in municipal areas other than Bangkok, 55.1 % and outside municipal areas in other parts of the country, 39.1%. Table 4 shows the prevalence of vitamin D insufficiency at 25(OH)D < 75 nmol/L and 25(OH) < 50 nmol/L by geographical regions and ge Individuals lived in Bangkok had the highest prevalence of vitamin D insufficiency, whereas people in the northeast had the lowest prevalence. After controlling for covariates in multiple linear regression analysis, the results showed that low serum 25(OH)D levels were associated with being female, younger age, urban and Bangkok (Table 5).

**Table 1:** Baseline characteristics of the population studies

Characteristics	Men	Women	Total
		
	(*n *= 1,321)	(*n *= 1,320)	
Age (years)	39.6 ± 0.5	41.0 ± 0.4	40.3 ± 0.3

BMI (kg/m^2^)	22.7 ± 0.2	24.4 ± 0.2*	23.6 ± 0.1

rural	71.5%	70.2%	70.8%

urban	28.5%	29.8%	29.2%

Religion:			

Muslim	2.5%	3.9%	3.2%

non Muslim	97.5%	96.1%	96.8%

Region:			
Bangkok	8.8%	9.4%	9.1%

Central	24.4%	24.9%	24.6%

North	18.3%	18.4%	18.3%

Northeast	34.9%	33.5%	34.2%

South	13.6%	13.8%	13.7%

Values are mean ± SE or percentage

* Significantly different from the men (*p *< 0.001)

**Table 2:** Duration of sunshine and mean serum vitamin D levels according to geographical region and gender

Regions	Duration of sunshine (hours/day)	25(OH)D (nmol/L)		
		
		Men	Women	Total
Bangkok	4.7 - 9.1	69.0 ± 0.6	61.1 ± 1.3*	64.8 ± 0.7***

Central	4.1 -8.5	86.5 ± 1.7**	73.0 ± 1.1*, **, ***	79.5 ± 1.1**, ***

North	3.6 - 8.1	88.5 ± 1.7**	75.1 ± 1.8*, **	81.7 ± 1.2**

Northeast	3.5 -8.0	87.8 ± 1.3**	*76.7 *± 1.1*, **	82.2 ± 0.8**

South	2.0 - 8.8	87.7 ± 2.9**	69.5 ± 0.8*, **, ***	78.3 ± 1.3**, ***

Total	2.0 - 9.1	85.9 ± 1.1	73.0 ± 0.8*	79.3 ± 0.8

Values are range or mean ± SE

*Significantly different from men (*p *< 0.001), ** significantly different from Bangkok (*p *< 0.001), *** significantly different from Northeast (*p *< 0.05)

**Table 3:** Mean serum 25(OH)D levels between gender by age, municipal area, BMI and religion

Variables		25(OH)D (nmol/L)			
		
		Men	Women	*p*	Total
Age (years)	15 - 29	79.3 ± 1.3	69.3 ± 1.1	<0.001	74.4 ± 0.9
	
	30 - 44	89.1 ± 1.7*	70.7 ± 1.2 ^a^	<0.001	79.9 ± 1.1*,, a
	
	45 - 59	86.5 ± 1.5*,	75.3 ± 1.2*,	<0.001	80.6 ± 1.0*
	
	60 - 69	90.7 ± 1.3*	80.2 ± 1.1*	<0.001	85.1 ± 1.0*
	
	70 - 79	95.0 ± 1.4*	83.8 ± 1.7*	<0.001	88.6 ± 1.2* ^a^
	
	> 80	96.9 ± 1.5*	80.7 ± 1.7*	<0.001	88.2 ± 1.4* ^a^

Municipal area	rural	88.9 ± 1.1	75.8 ± 0.8	<0.001	82.3 ± 0.6
	
	urban	78.4 ± 1.6**	66.6 ± 1.1**	<0.001	72.3 ± 1.3**

BMI (kg/m2)	≥ 25	84.1 ± 1.9	73.7 ± 1.1	<0.001	77.5 ± 1.1
	
	< 25	86.5 ± 1.1	72.6 ± 0.9	<0.001	80.3 ± 0.8***

Religion	Muslim	81.9 ± 5.2	61.1 ± 4.2	= 0.001	69.0 ± 4.4
	
	non Muslim	86.0 ± 10****	73.5 ± 0.7****	<0.001	79.7 ± 0.7****

*Significantly different from age 15 -29 years (*p *< 0.001), **significantly different from rural (*p *< 0.001), *** significantly different from BMI ≥ 25 (*p *< 0.001), **** significantly different from Muslim (*p *< 0.05) ^a^significantly different from age > 80 years

**Table 4:** Prevalence of vitamin D insufficiency by geographical region and gender

Regions	Age, yrs (range)	Serum 25(OH)D levels					
		
		< 75 nmol/L			< 50 nmol/L		
		
		Men	Women	Total	Men	Women	Total
Bangkok	15 - 93	66.7%	75.5%	64.6%	10.8%	24.2%	14.3%

Central	15 - 91	36.2%	59.2%	43.1%	2.1%	11.4%	6.5%

North	15 - 98	27.9%	50.8%	39.1%	0.9%	6.5%	4.3%

Northeast	15 - 91	25.1%	51.0%	34.2%	0.1%	3.7%	2.8%

South	15 - 92	29.4%	65.8%	43.8%	1.5%	12.9%	6.3%

Total	15 - 98	32.6%	57.3%	45.2%	1.9%	9.3%	5.7%

**Table 5:** Independent variables for serum 25(OH)D levels by multiple regression analysis

Independent variables	Regression coefficient	SE	*p*
gender	12.97	0.957	< 0.001

Age	0.28	0.019	< 0.001

Urban	-6.47	1.04	< 0.001

BMI	-0.08	0.094	0.422

Muslim	-0.82	2.358	0.733

Region^a^:			

Bangkok	-12.01	1.519	<0.001

Central	-1.73	1.543	0.279

North	-0.69	1.575	0.664

South	-3.32	1.648	0.060

^a^reference group: Northeast

## Discussion

Our study represents the first large-scale examination of vitamin D status in the Thai population. Despite the fact that Thailand is located near the equator, a sizable proportion of Thais have inadequate vitamin D status. When using a 25(OH)D threshold of 75 nmol/L, nearly half of Thais are vitamin D insufficient. When a lower threshold of 50 nmol/L was used, the prevalence of vitamin D insufficiency was found to be more than 10% in Bangkok, which is as high as the prevalence of diabetes in Thailand [5]. Studies examining vitamin D status in the tropics are scarce, but have mostly demonstrated similarly low vitamin D status. For example, even in the sunniest areas like Saudi Arabia, the United Arab Emirates, Australia, Turkey, India and Lebanon, a high prevalence of vitamin D insufficiency has been reported in 30 to 50% of children and adults, with 25(OH)D levels under 50 nmol/L [6-10]. On the other hand, people living near the equator who are exposed to sunlight without sun protection have robust levels of 25(OH)D, well above 75 nmol/L [11]. Taken together, this suggests that low vitamin D status is not an uncommon problem even in countries that receive abundant sunshine. Despite this, outdoor sun exposure can be limited and is likely to be the main contributing factor.

The mean serum 25(OH)D levels in Thais seem relatively high when compared to those reported in various countries in the West [3,12], Middle East [13] and Asia [14]. This might be caused by a higher exposure to sunshine all year round, since the latitude of Thailand is more southerly (closer to the equator) than the countries studied. Nevertheless, differences in vitamin D status were found between regions in Thailand; subjects residing in the southern parts of the country, women in particular, generally had lower serum 25(OH)D concentrations than those residing in the northern region. This finding conflicts with the belief that vitamin D status decreases with increasing latitude. However, our results are in agreement with a European study which showed a positive relationship between serum 25(OH)D and northern latitude [4]. This finding could be explained by the common use of cod liver oil and vitamin supplements in many northern European countries; while people in southern Europe typically have more skin pigmentation (with consequently less vitamin D production) and may prefer shade instead of sunshine. An explanation for this observation in Thailand might be regional differences in religion. Southern Thailand has a much higher percentage of Muslims, and the clothing style of Muslim women generally allows for greater body coverage. In addition, most northern Thai people are agricultural. Working in the fields and spending more time outdoors in the sunshine probably accounts for much of this difference.

Both lifestyle and environmental factors are important determinants of serum 25(OH)D concentration because of their relationship to ultraviolet exposure. In the present study, a difference in vitamin D status between populations in rural and urban areas was clearly demonstrated. Lower vitamin D levels in the urban populations were evident in almost all geographical regions of Thailand. Although a number of studies have investigated the vitamin D status of urban or rural residents, the disparity in vitamin D status between rural and urban populations has been investigated less often; but existing studies have generally shown lower vitamin D reserves among urban populations [15-17]. A number of factors may be causally related to lower vitamin D status associated with urbanization. Besides lifestyle factors, which may preclude adequate outdoor sun exposure, it is also likely that air pollution may have a contributory role. Tropospheric ozone is a common urban air pollutant and an efficient absorber of ultraviolet radiation [18]. The phenomenon is likely to be more marked in big cities, and may partially explain why residents of Bangkok, the largest city in Thailand, had the lowest 25(OH)D concentrations.

Lower vitamin D status has been demonstrated to be more prevalent with advancing age in most studies [19-22]; this may be caused by less sun exposure and the decreased ability of the skin to produce vitamin D [23]. In contrast, we demonstrated in the present study that vitamin D levels unexpectedly became higher with increasing age. Younger age, rather than older, was an independent risk factor for inadequate vitamin D status. The phenomenon of higher vitamin D levels with advancing age was also observed in both sexes, making it less likely that the observation was simply a chance finding. Although most studies have demonstrated lower vitamin D levels with advancing age, such findings have been predominantly generated from studies of populations residing in temperate geographical locations. There are only limited data on this issue for countries in the tropics. It was found in a study of postmenopausal women in Malaysia that vitamin D levels do not decrease with age [24]. Likewise, 25(OH)D levels remain more or less constant from age 20 to more than age 60 in Iranian men [25]. It is therefore conceivable that despite the decreased dermal synthesis of vitamin D in the elderly, the abundant sunlight may overcome this disadvantage, given that sun exposure is not limited. The elderly in Thailand, after retirement, may have more leisure time and spend more time in the sun. On the other hand, it is also likely that the increased use of sunblock by younger people may be partly accountable for their lower vitamin D status compared to the older population.

## Conclusion

Vitamin D insufficiency is highly prevalent in the general adult population in Thailand. Vitamin D status is better in northern than in southern regions of the country. Low serum 25(OH)D levels were associated with being female, younger age, living in urban and Bangkok.

## Authors' contributions

BO and WA designed research. LC and WA conducted research and performed the statistical analysis. LC and BO drafted the manuscript. All authors read and approved the final manuscript

## Competing interests

The authors declare that they have no competing interests.

## Pre-publication history

The pre-publication history for this paper can be accessed here:

http://www.biomedcentral.com/1471-2458/11/853/prepub
